# A Novel Constitutively Active *c*.98*G* > *C*, p.(R33P) Variant in *RAB11A* Associated with Intellectual Disability Promotes Neuritogenesis and Affects Oligodendroglial Arborization

**DOI:** 10.1155/2023/8126544

**Published:** 2023-08-07

**Authors:** Yumi Tsuneura, Taeko Kawai, Keitaro Yamada, Shintaro Aoki, Mitsuko Nakashima, Shima Eda, Tohru Matsuki, Masashi Nishikawa, Koh-ichi Nagata, Yasushi Enokido, Hirotomo Saitsu, Atsuo Nakayama

**Affiliations:** ^1^Department of Cellular Pathology, Institute for Developmental Research, Aichi Developmental Disability Center, Kasugai 486-0392, Japan; ^2^Department of Pediatric Neurology, Central Hospital, Aichi Developmental Disability Center, Kasugai 486-0392, Japan; ^3^Department of Biochemistry, Hamamatsu University School of Medicine, Hamamatsu 431-3192, Japan; ^4^Department of Molecular Neurobiology, Institute for Developmental Research, Aichi Developmental Disability Center, Kasugai 486-0392, Japan; ^5^Department of Neurochemistry, Nagoya University Graduate School of Medicine, Nagoya 466-8560, Japan

## Abstract

Whole exome sequencing/whole genome sequencing has accelerated the identification of novel genes associated with intellectual disabilities (ID), and *RAB11A* which encodes an endosomal small GTPase is among them. However, consequent neural abnormalities have not been studied, and pathophysiological mechanisms underlying the ID and other clinical features in patients harboring *RAB11A* variants remain to be clarified. In this study, we report a novel *de novo* missense variant in *RAB11A*, NM_004663.5: *c*.98*G* > *C*, which would result in NP_004654.1: p.(R33P) substitution, in a Japanese boy with severe ID and hypomyelination. Biochemical analyses indicated that the RAB11A-R33P is a gain-of-function, constitutively active variant. Accordingly, the introduction of the RAB11A-R33P promoted neurite extension in neurons like a known constitutively active variant Rab11A-Q70L. In addition, the RAB11A-R33P induced excessive branching with thinner processes in oligodendrocytes. These results indicate that the gain-of-function RAB11A-R33P variant in association with ID and hypomyelination affects neural cells and can be deleterious to them, especially to oligodendrocytes, and strongly suggest the pathogenic role of the RAB11A-R33P variant in neurodevelopmental impairments, especially in the hypomyelination.

## 1. Introduction

RAB11 is a small GTPase which alternates between a GTP-bound active form and a GDP-bound inactive form like other members of the Ras GTPase superfamily and belongs to the large RAB subfamily that acts as essential regulators of vesicular trafficking. Localized to the surface of recycling endosomes and post-Golgi vesicles that distribute various membrane proteins and membrane itself to the plasma membrane, RAB11 has important roles in the supply of receptor and adhesion proteins as well as local membrane dynamics [1]. As a result, RAB11 is involved in diverse cell activities including cell polarization, ciliogenesis, cytokinesis, neuritogenesis, and oogenesis [2–7]. In mammalian neurons, active RAB11 upregulates axon extension, dendritic arborization, and dendritic spine formation [6, 8, 9].

To date, six distinct *de novo* missense (DNM) variants in *RAB11A* have been reported in association with developmental disorders [10–12]. Some of the predicted variant proteins have been studied about their structural and biochemical properties [13]. It, however, remains to be unveiled how these variant proteins affect neurodevelopment. We here report a novel DNM *RAB11A* variant, NM_004663.5: *c*.98*G* > *C* (NP_004654.1: p.(R33P)), identified in a boy with severe intellectual disability (ID) and hypomyelination. Biochemical and neurobiological analyses revealed that the R33P variant is constitutively active, and the expression of the variant promoted neurite extension in neurons. Furthermore, the variant induced abnormal arborization in oligodendrocytes. These results provide the first evidence that a gain-of-function DNM *RAB11A* variant causes neuronal and oligodendroglial abnormalities and strongly suggest that it is a disease-associated variant having deleterious effects on neurodevelopment.

## 2. Materials and Methods

### 2.1. Human Subject

The Japanese boy was referred for neurodevelopmental examination to the Central Hospital, Aichi Developmental Disability Center (Kasugai 480, Aichi, Japan) due to developmental delay. Physical examinations, laboratory examinations of blood and urine samples, brain MRI, and genetic analyses were performed for a diagnostic purpose. An investigation which collects existing clinical and genetic data and publishes them in an anonymized form was exempted from the approval by the Ethics Committee of Aichi Developmental Disability Center.

### 2.2. Whole Exome Sequencing (WES)

After receiving a written informed consent from the parents, genomic DNA of the patient and his parents was extracted from blood leukocytes. Genomic DNA of the patient was captured using an xGen Exome Research Panel kit (IDT, Coralville, IA, USA) and sequenced by a NextSeq 500 (Illumina) with 75-bp paired-end reads. Exome data processing, variant calling, variant annotation, and filtering were performed as previously described [14]. Briefly, we focused on rare nonsynonymous variants with minor allele frequencies below 1% in the three public databases and our in-house exome data. Then, we searched variants in our custom-made causative gene panel for monogenic disorders including *RAB11A*. Candidate variants were confirmed by Sanger sequencing using an ABI 3130xl Genetic Analyzer (Applied Biosystems, Foster City, CA, USA). The biological parentage was confirmed by analyzing 10 microsatellite markers (data not shown). Experimental protocols were approved by the Institutional Review Board of Hamamatsu University School of Medicine as approval #17-163.

### 2.3. Animals

Pregnant ICR mice were purchased from Japan SLC (Hamamatsu, Japan). All mice were handled in accordance with an experimental protocol approved by the Animal Care and Use Committee in the Institute for Developmental Research (approval number: 2019-013). We used the ARRIVE guidelines 2.0: author checklist to report the animal experiment [15].

### 2.4. Antibodies

Antibodies used in this study were as follows: anti-green fluorescent protein (GFP) (Aves, Davis, CA, USA, GFP-1010), anti-microtubule associated protein 2 (MAP2) (Cell Signaling Technology, Danvers, MA, USA, 8707 T), anti-phosphorylated neurofilament (Covance, Princeton, NJ, USA, SMI-312R), anti-myelin basic protein (MBP) (Santa Cruz, Dallas, TX, USA, sc-13914), anti-RAB11A (Proteintech, Rosemont, IL, USA, 20229-1-AP), anti-RAB11B (Proteintech, 19742-1-AP), anti-RAB5A (GeneTex, Irvine, CA, USA, GTX109665), anti-RAB7 (Cell Signaling Technology, 95746), anti-*β*-actin (FUJIFILM Wako Pure Chemical Corporation, Osaka, Japan, 013-24553), Alexa 488-conjugated goat anti-chicken IgY (Thermo Fisher Scientific, Waltham, MA, USA, A-11039), Alexa 568-conjugated goat anti-rabbit IgG (Thermo Fisher Scientific, A-11036), Alexa 568-conjugated goat anti-mouse IgG (Thermo Fisher Scientific, A-11031), Alexa 568-conjugated donkey anti-goat IgG (Abcam, Cambridge, UK, ab175704), and peroxidase-conjugated anti-mouse or -rabbit secondary antibody (Kirkegaard and Perry Laboratories Inc., Gaithersburg, MD, USA, 047-1806, 074-1506).

### 2.5. Plasmid

A full-length *RAB11A* cDNA (NM_004663.5) was cloned into a pTriEx-4 vector (Merck, Darmstadt, Germany) using 5× In-Fusion Snap Assembly Master Mix (Takara, Kusatsu, Japan, ST2317). pEF-FLAG-Rab11A, -Rab11A-Q70L, and -Rab11A-S25N were kindly provided by Dr. Fukuda at Tohoku University. A cDNA for the RAB11A-R33P variant was generated by mutagenesis using PrimeSTAR Max DNA Polymerase (Takara, R045A). A lentivirus plasmid (pLCBA) carrying chicken beta actin (CBA) promoter for exogenous gene expression was developed by modifying pLL3.7 (Addgene, Watertown, MA, USA, 11795). We substituted the region from the U6 promoter to EGFP for the CBA promoter and used it to construct pLCBA-RAB11A-T2A-EGFP and its variants.

### 2.6. Biochemical Assays

GTP-hydrolysis and intrinsic GTP/GDP-exchange activities were assessed as previously described [16]. His-tagged Rab11A and its variants were prepared according to the manufacturer's instructions. GTP-hydrolysis activities were measured using GTPase-Glo assay kit (Promega, Madison, WI, USA). GTP/GDP-exchange assay was performed by monitoring fluorescent decay of GDP analog, methylanthraniloyl-GDP (^mant^GDP, Sigma-Aldrich) [17].

### 2.7. Lentivirus Preparation

Lentivirus vectors were prepared as described previously [18] with minor modifications. HEK293FT cells were transfected with pLCBA-RAB11A-T2A-EGFP or its variants together with psPAX2 and pCMV-VSV-G-RSV-Rev for packaging, using PEI MAX (25 kDa, Polysciences, Warrington, PA, USA). Forty-eight hours after transfection, the virus-containing medium was collected and centrifuged to remove debris. The supernatant was centrifuged at 6000 × g overnight, and the pellet containing lentiviruses was suspended in a neurobasal medium (Thermo Fisher Scientific).

### 2.8. Preparation and Morphological Analyses of Hippocampal Neurons

Mouse hippocampal neurons were cultured as described previously [18]. Briefly, neurons were prepared from the hippocampi of mouse embryos at embryonic day 16.5 and were plated at 2 × 10^4^ cells/cm^2^ on poly-L-lysine coated coverslips. The neurons were cultured in a neurobasal medium containing 2% B27 and Glutamax-I (Thermo Fisher Scientific). At 0 day in vitro (DIV), the neurons were infected with lentivirus vectors at a multiplicity of infection (MOI) of 0.5. The infected neurons were fixed at 4 DIV with 4% paraformaldehyde (PFA) for 10 min at room temperature and then incubated in 0.1% Triton X-100 for 5 min. After incubation in blocking buffer (5% BSA in PBS) for 1 h, primary antibodies diluted with blocking buffer were added and incubated at 4°C overnight. The antibodies were diluted as follows: anti-GFP (1 : 2,000), anti-SMI-312 (1 : 1,000), and anti-MAP2 (1 : 1,000). The neurons were washed with PBS and incubated with secondary antibodies in blocking buffer at room temperature for 2 h. The secondary antibodies were diluted as follows: Alexa 488-conjugated goat anti-chicken IgY (1 : 1,000), Alexa 568-conjugated goat anti-rabbit IgG (1 : 1,000), and Alexa 568-conjugated goat anti-mouse IgG (1 : 1,000). The neurons on the coverslip were mounted using VECTASHIELD mounting medium with DAPI (Vector Laboratories, Burlingame, CA, USA) and were observed with Zeiss LSM 880 confocal microscopy system (Carl Zeiss, Oberkochen, Germany). The lengths of SMI-312-positive axons were measured using MetaMorph software (Molecular Devices, San Jose, CA, USA). Analyses of MAP2-positive dendrites were performed on reconstructed 3D images using the Filament Tracer function in Imaris software (Bitplane AG, Zurich, Switzerland).

### 2.9. Preparation and Morphological Analysis of Oligodendrocytes

Oligodendrocyte precursor cells (OPCs) were isolated from postnatal day 8-9 mouse cortices and hippocampi by immunopanning with antibodies against platelet-derived growth factor receptor- (PDGFR-) *α* (558774, BD PharMingen, San Diego, CA, USA), as previously described [19–21]. Shortly, mouse brain tissues were enzymatically dissociated with papain (Worthington Biochemical, Lakewood, NJ, USA, 9 U/mL) for 30 min at 37°C, and positively immunopanned for PDGFR-*α* after a depletion of microglia with BSL1 (Vector Laboratories, 2.5 *μ*g/mL). OPC purity levels were determined by immunostaining for PDGFR-*α* and Olig2 and were confirmed to be 97% or more. Cells were plated at 2–5 × 10^4^ cells/cm^2^ and grown in serum-free media, but with the addition of 1% N21-MAX Media Supplement (R&D Systems, Minneapolis, MN, USA). Platelet-derived growth factor-AA (PDGF; FUJIFILM Wako Pure Chemical Corporation, 10 ng/mL) and neurotrophin-3 (NT-3; FUJIFILM Wako Pure Chemical Corporation, 5 ng/mL) were added to the media to proliferate OPCs, whereas PDGF and NT-3 were removed, and 1% fetal bovine serum, triiodothyronine (T3) (40 ng/mL; Sigma-Aldrich) and ciliary neurotrophic factor (CNTF; 10 ng/mL, FUJIFILM Wako Pure Chemical Corporation) were added to the differentiation media for oligodendrocyte differentiation. For the introduction of the various RAB11As, OPCs were infected with lentivirus vectors at a MOI of 0.5 when the media is replaced to the differentiation media. Cultured cells were fixed and subjected to immunocytochemistry in a similar way as neurons were processed. Applied antibodies were diluted as follows: anti-myelin basic protein (MBP) (1 : 200), anti-GFP (1 : 2,000), Alexa 488-conjugated goat anti-chicken IgY (1 : 500), and Alexa 568-conjugated donkey anti-goat IgG (1 : 500). The stained oligodendrocytes were morphologically analyzed using the Sholl plugin for ImageJ (NIH, Bethesda). Fragmented each binary picture of the process meshwork visualized by MBP staining in oligodendrocytes (*n* = 25 in each condition) was subjected to Sholl analysis [22, 23]. To distinguish the cytoplasmic process structure from the background, spread-out membranes, the trainable weka segmentation V2.2.1 plugin for ImageJ was used in the standardized training settings. The total numbers of process intersections per cell and the radial lengths of the outermost rings with process intersections in each cell were analyzed in the following settings: starting radius = 15 *μ*m; radius step size = 15 *μ*m.

### 2.10. Western Blotting

Cultured cells were washed three times with ice-cold PBS and dissolved in lysis buffer containing 62.5 mM Tris-HCl pH 6.8, 2% (*w*/*v*) SDS, 2.5% (*v*/*v*) 2-mercaptoethanol, and 5% (*v*/*v*) glycerol. Protein concentrations were quantified by using the BCA method (Pierce BCA protein assay kit; Thermo Fisher Scientific). The protein samples were subjected to electrophoresis (5 *μ*g protein/lane) on SDS-polyacrylamide gels and electroblotted onto polyvinylidene difluoride membranes (Millipore, Burlington, MA, USA). The membranes were blocked with 5% globulin-free BSA (FUJIFILM Wako Pure Chemical Corporation) in TTBS (20 mM Tris-HCl, pH 7.4, 150 mM NaCl, and 0.1% Tween 20), and incubated with primary antibodies overnight at 4°C. The dilution conditions of primary antibodies were as follows: anti-RAB11A (1 : 1,000), anti-RAB11B (1 : 1,000), anti-RAB5A (1 : 1,000), anti-RAB7 (1 : 1,000), and anti-*β*-actin (1 : 10,000). Then, the membranes were incubated with peroxidase-conjugated secondary antibodies (1 : 5,000) in TTBS. Immunoreactivity was detected as chemiluminescence signals with Western BLoT Quant HRP Substrate (Takara), and was digitized with ImageQuant LAS 4000 (GE Healthcare, Chicago, IL, USA).

### 2.11. Statistical Analysis

Statistical analyses were performed with GraphPad Prism7J (GraphPad Software, San Diego, CA, USA). A one-way analysis of variance (ANOVA) followed by Tukey's multiple comparison tests was used for more than two groups.

## 3. Results

### 3.1. Clinical Features

A Japanese boy was born normally at 39 weeks weighing 2,812 g (−0.45 SD), with a height of 50.0 cm (+0.47 SD) and head circumference of 32.5 cm (−0.57 SD), as a first child of healthy, unrelated parents. There was no family history of neurodevelopmental disorders. His developmental milestones were mildly delayed: head control at 5 months of age and rolling over at 7 months of age.

He was referred to the Central Hospital at 11 months of age due to delayed development; he could not sit and pull oneself up at that point. At an initial visit, he showed no dysmorphic features or failure to thrive. He showed normal muscle tonus and normal tendon reflexes in the upper and lower limbs by physical examination. Ophthalmological examinations indicated no obvious abnormalities. Laboratory data, including the concentrations of thyroid stimulating hormone and free triiodothyronine 3 and 4, lactate and pyruvate levels, and amino acid profiles in blood, were normal. G-banded analysis showed a normal karyotype (46, XY). Subtelomere fluorescence in situ hybridization analysis and *PLP1* duplication/deletion analysis supplied no abnormal findings.

**Figure 1:**
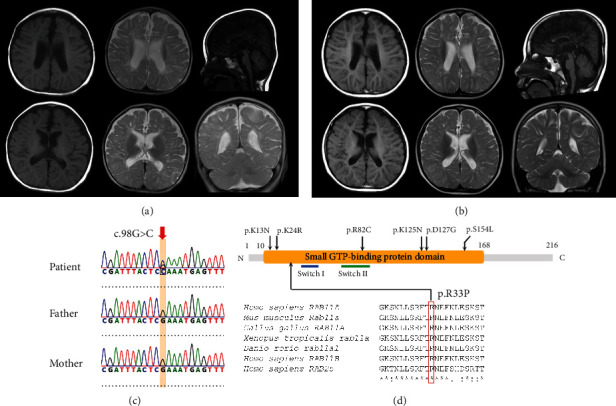
Neuroimaging and genetic analysis data on the patient. Brain images obtained at 1 year and 8 months old (a) and 5 years and 8 months old (b). Respective axial T1-weighted images (the top-left and bottom-left) and T2-weighted images (the top-center and bottom-center), a sagittal T1-weighted image (the top-right), and a coronal T2-weighted image (the bottom-right) are shown. All images indicate hypomyelination in the patient's brain. (c) Sanger sequencing validated *de novoc*.98*G* > *C* variant in *RAB11A*. Electropherograms from the patient and his parents are shown. (d) Schematic RAB11A structure displaying the small GTP-binding protein domain, and the switch I and II domains. Amino acid positions predicted to be substituted in individuals with intellectual disability/developmental delay reported previously are presented above, and the position putatively substituted in the present case is below. Aligned partial amino acid residues in various species RAB11A/Rab11a are shown below, and conserved Arg33 across species is boxed.

Thereafter, periodic brain MRI examinations were performed at 1 year and 8 months and 5 years and 8 months of age and revealed global hypomyelination, hypoplasia of the white matter with lateral ventricular enlargement, and hypoplasia of the corpus callosum (Figures 1(a) and 1(b)). His developmental milestones became severely delayed: crawling and sitting independently at 1 year old, standing with support at 1 year and 5 months old, and walking independently at 5 years old. At 6 years old, he had no problems in physical growth nor seizure episodes. He had severe intellectual disability without any meaningful words or expressions but had a friendly personality.

### 3.2. Genetic Analyses

At least 96.5% of the target RefSeq coding sequences were covered by 20 reads or more. Using WES data, we found a candidate variant, NM_004663.5: *c*.98*G* > *C*, in *RAB11A*, which would result in NP_004654.1: p.(R33P) (Figures 1(c) and 1(d)). The substituted Arg33 was evolutionarily conserved and located between the nucleotide-binding pocket and nucleotide-sensitive switch1 (Figure 1(d)). This variant was absent in our 218 in-house Japanese control exome data and public databases, including the Genome Aggregation Database (gnomAD, http://gnomad.broadinstitute.org/) and the 38KJPN (https://jmorp.megabank.tohoku.ac.jp), and predicted to be deleterious by multiple *in silico* pathogenicity prediction tools (Table 1). Sanger sequencing revealed that this variant occurred *de novo* (Figure 1(c)); therefore, this variant was classified as likely pathogenic according to the American College of Medical Genetics Standards and Guidelines (PS2, PM1, PM2, and PP3). Moreover, we examined possible pathogenic copy number variants (CNVs) using WES data with the eXome-Hidden Markov Model (XHMM) [24] and the methods developed by Nord et al. [25]; however, no possible candidate CNVs were found in this case. Thus, we considered that the DNM *RAB11A* variant was likely to be causative in this case.

### 3.3. RAB11A-R33P Is a Gain-of-Function, Constitutively Active Variant

**Figure 2:**
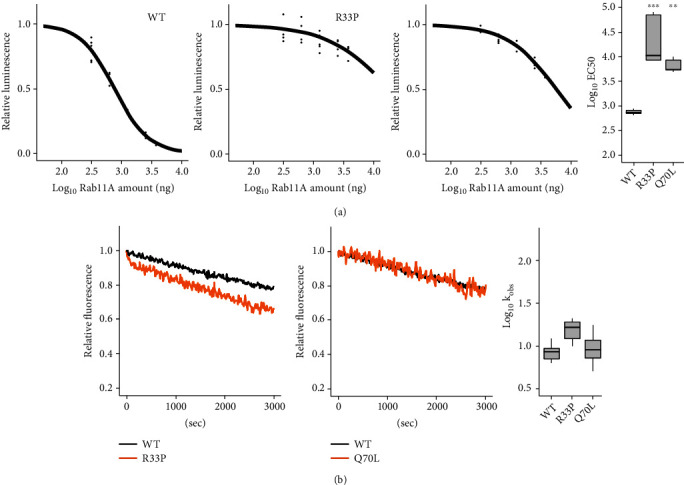
Biochemical activity of RAB11A-R33P. (a) Measurement of GTP-hydrolysis activity. The activities were assessed by measuring residual amounts of GTP indicated as luminescence intensities with GTPase Glo assay kit. EC50 (half maximal effective concentration) values for each RAB11A wild type (WT), -R33P, and -Q70L were estimated from respective sigmoidal fitting curves and were shown as a box plot at the far right (*n* = 6). ^∗∗^*p* < 0.01, ^∗∗∗^*p* < 0.001 (versus RAB11A WT). (b) Measurement of intrinsic GTP/GDP exchange activity. RAB11A wild type (WT), -R33P, and -Q70L were preloaded with fluorescent ^mant^GDP and incubated with nonhydrolysable GTP analog. ^mant^GDP dissociation rates were determined by monitoring fluorescent decay that is caused by the replacement of fluorescent ^mant^GDP by GTP. Observed rate constant (kobs (×10^−5^ s^−1^)) for each RAB11A was calculated and shown as a box plot in the right (*n* = 8).

To reveal the effects of the R33P substitution on the biochemical activities of RAB11A, we first measured the GTP-hydrolysis activities of the R33P variant, together with wild type (WT) and a constitutively active Rab11A-Q70L variant for comparison. The Rab11a-Q70L and constitutively inactive Rab11a-S25N described below were developed as tools to study the physiological role of Rab11 and are unrelated to known human *RAB11A* variants [26]. The activities, which were indicated as decreasing rates of luminescence, of the R33P and Q70L variants were significantly lower than that of WT, revealing that the R33P variant is prone to stay in a GTP-bound active form like the Q70L variant (Figure 2(a)). Then, we examined intrinsic GTP/GDP exchange activities by monitoring fluorescent decay that is caused by the replacement of fluorescent ^mant^GDP by GTP and found that the activity of the R33P was slightly, but not significantly high (*p* = 0.0547) compared with that of WT (Figure 2(b)). Taken together, these results indicated that, like the Rab11A-Q70L, the RAB11A-R33P is prone to be a GTP-bound active form and a gain-of-function, constitutively active variant.

### 3.4. RAB11A-R33P Promotes Neurite Extension in Neurons

**Figure 3:**
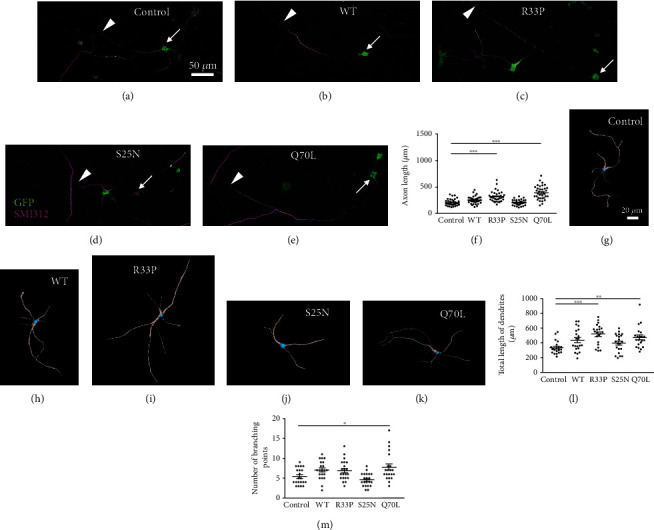
Effect of RAB11A-R33P expression on neuronal morphology. (a–e) Representative images of primary cultured hippocampal neurons labeled with anti-GFP (green) and antiphosphorylated neurofilament (SMI-312, magenta). Hippocampal neurons infected with lentiviral EGFP alone (a) or EGFP-RAB11A WT (b), -RAB11A-R33P (c), -Rab11A-S25N (d), and -Rab11A-Q70L (e) at 0 DIV were analyzed at 4 DIV. Cell bodies and axon terminals are indicated by arrows and arrowheads, respectively. (f) A graph showing quantified axonal lengths in each group. Values (*n* = 36) are indicated as dots and the mean ± SEM in each group is presented as bars. ^∗∗∗^*p* < 0.001. (g–k) Representative reconstructed 3D images of primary cultured hippocampal neurons infected with lentiviral vectors as in (a–e) and labeled with anti-GFP and anti-MAP2. Cell bodies stained with anti-GFP alone are blue-colored, and dendrites stained with anti-MAP2 are gray-colored in all images. Graphs showing quantified total lengths of dendrites (l) and numbers of branching points of dendrites (m) in each group. Values (*n* = 21) are indicated as dots and the mean ± SEM in each group is shown as bars. ^∗^*p* < 0.05, ^∗∗^*p* < 0, 01, ^∗∗∗^*p* < 0.001.

A previous study indicated that the constitutively active Rab11A-Q70L promoted axon extension, while a constitutively inactive Rab11A-S25N did not [6]. Therefore, we reasoned that the R33P variant, which was biochemically constitutively active, might also affect axon extension in neurons. As shown in Figures 3(a)–3(e), the axons of cultured hippocampal neurons expressing the R33P (Figure 3(c)) and the Q70L (Figure 3(e)), respectively, looked longer than those of the control neurons (Figure 3(a)) and neurons expressing WT (Figure 3(b)) and the S25N (Figure 3(d)). Quantification confirmed that the mean axon lengths of neurons with respective R33P and Q70L expression were significantly longer than that of the control (Figure 3(f)), whereas the overexpression of WT or the S25N has no significant effects on axon extension. The R33P promoted dendrite extension as well like the Q70L (Figures 3(g)–3(l)); however, the R33P showed less obvious effects on dendritic branching while the Q70L significantly enhanced it (Figure 3(m)) as reported previously [9]. These results indicated that the R33P promotes neurite extension in agreement with the fact that it is biochemically constitutively active.

### 3.5. RAB11A-R33P Induces Abnormal Arborization in Oligodendrocytes

**Figure 4:**
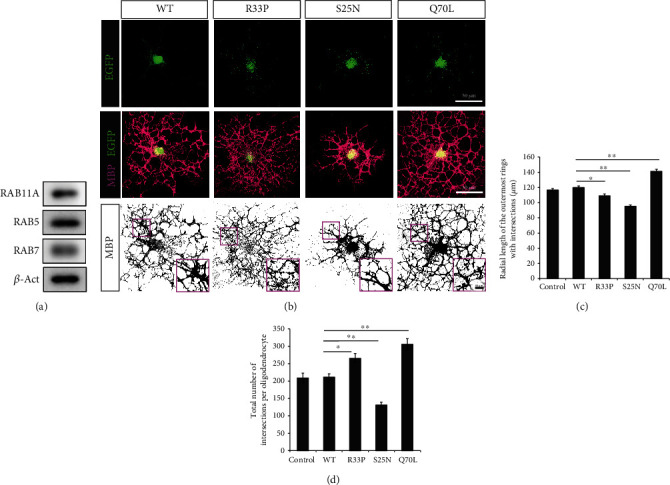
The expressions of RABs in oligodendrocytes and effect of RAB11A-R33P expression on oligodendrocyte morphology. (a) RAB11A, 5, and 7 are expressed in cultured mouse oligodendrocytes. Western blotting of cultured oligodendrocyte lysates was performed, and signal bands confirming the expressions of RAB11A, 5, and 7 are shown. *ß*-Actin was used as a loading control. (b) Representative images of primary cultured oligodendrocytes expressing RAB11A WT, -R33P, -S25N, or -Q70L variants. At 96 h, after the induction of differentiation, cells were stained with antibodies against GFP and MBP. The top: representative images of oligodendrocytes expressing EGFP-tagged RAB11A WT and variants. The middle: representative images of oligodendrocytes stained with MBP merged with EGFP images. MBP images display arborized cytoplasmic processes in each oligodendrocyte. The bottom: binary images of MBP staining visualizing detailed arborization. The insets show high-power magnification views of boxed areas. Scale bars: 50 *μ*m (the top and middle), 10 *μ*m (the bottom inset). (c, d) Graphs presenting Sholl analysis data on oligodendrocytes expressing the various RAB11A. The mean of the total numbers of intersections in each oligodendrocyte (c), and the mean of the radial lengths of the outermost rings intersecting the oligodendroglial process (d) are shown (*n* = 25 in each group). Oligodendrocytes infected with an empty EGFP vector were used as control. ^∗^*p* < 0.05; ^∗∗^*p* < 0.01 versus RAB11A WT.

The promoted neurite extension caused by the constitutively active RAB11A-R33P in neurons may disturb normal development of the nervous system; however, this neuronal abnormality seemed to explain not enough about the severe hypomyelination observed in the present case. Therefore, we next reasoned that RAB11A would be expressed and have important roles in oligodendrocytes whose functions highly depend on the plasma membrane dynamics and that the RAB11A-R33P would affect the structures of oligodendrocytes. As expected, RAB11A was expressed together with other endosomal RABs, RAB5, and RAB7, in cultured oligodendrocytes (Figure 4(a)). Oligodendrocytes expressing the RAB11A-R33P looked to have thinner processes with excessive branching but without the expansion of the areas covered by the processes, whereas those expressing WT showed no obvious morphological alterations when compared with the control oligodendrocytes to which an empty EGFP vector was introduced (Figure 4(b) and not shown). The Sholl analysis indicated that the mean of the total numbers of intersections, an indicator of branching complexity, in oligodendrocytes expressing the R33P was significantly greater than that in oligodendrocytes expressing WT (Figure 4(c)). It was also revealed that the mean of the radial lengths of the outermost rings with intersections, which represents the size of the area covered by the cytoplasmic processes, in oligodendrocytes expressing the R33P was rather smaller than that in oligodendrocytes expressing the WT (Figure 4(d)). Intriguingly, the Q70L induced increased branching of and expanded areas covered by the cytoplasmic processes, while the S25N induced decreased branching of and shrunk areas covered by the processes (Figures 4(b)–4(d)). These data indicated that the constitutively active Q70L accelerated the growth of oligodendrocytes while the constitutively inactive S25N decelerate it, and the R33P induced unbalanced excessive branching of the processes without the expansion of their coverage areas.

## 4. Discussion

We identified a novel DNM variant, NM_004663.5: *c*.98*G* > *C* (NP_004654.1: p.(R33P)) in *RAB11A* in a boy with severe ID and hypomyelination otherwise lacking apparent brain anomalies. Biochemical analyses of the RAB11A-R33P revealed that the protein was prone to be in a GTP-bound active state and was considered a constitutively active variant. Accordingly, the R33P variant promoted axon and dendrite extension in cultured hippocampal neurons like a known constitutively active variant, Rab11A-Q70L. Furthermore, the R33P induced excessive branching and fine thinner processes in cultured oligodendrocytes. These findings, especially novel cell biological findings, suggested the pathogenicity of the *RAB11A* variant, *c*.98*G* > *C* (p.(R33P)), in ID and hypomyelination via inducing neuronal and oligodendroglial abnormalities.

Hamdan et al. found the first disease-associated *RAB11A* variant, *c*.244*C* > *T* (p.(R82C)) in a cohort of developmental and epileptic encephalopathy (DEE) patients, and identified additional three variants, *c*.71*A* > *G* (p.(K24R)), *c*.461*C* > *T* (p.(S154L)), and *c*.39*A* > *C* (p.(K13N)) in individuals with ID/global developmental delay (GDD)/unspecified developmental disorder without epilepsy in their cohort and an external dataset (Table 2) [10, 11]. Bertoli-Avella et al. reported two additional *RAB11A* variants, *c*.375*G* > *T* (p.(K125N)) and *c*.380*A* > *G* (p.(D127G)) in individuals with ID/GDD (Table 2) [12]. Thus, although the primary case (case #2 in Table 2) was identified through a study on the DEE cohort, epilepsy seems not the common feature in individuals harboring DNM *RAB11A* variants. Rather, hypomyelination and hypogenesis/agenesis of the corpus callosum without apparent cortical anomalies are considered the common phenotypes of them when case #7 is excluded (Table 2). Therefore, we deduce that the individuals harboring DNM *RAB11A* variants are characterized as ID/DD associated with impaired white matter development otherwise lacking common abnormal features, although their definite clinical characteristics must be determined based on the further accumulation of cases.

**Figure 5:**
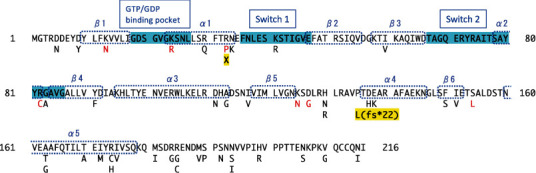
Summary of disease-associated and nondisease-associated RAB11A variants. RAB11A amino acid residue (NP_004654.1) is presented, and the regions of major functional domains (GTP/GDP binding pocket, switch1, and switch2) and those taking secondary structures (*α* helix 1-5 and *β* strand 1-6) are indicated with blue markings and dashed lined boxes, respectively. Below the residue, disease-associated variants (red symbols) and putative nondisease-associated variants (black symbols) are shown. One nonsense variant and one deletion variant resulting in a premature stop codon among the nondisease-associated variants are marked in yellow. The nondisease-associated variants were extracted from gnomAD v3.1.2 (nonneuro) and v2.1.1 (nonneuro) datasets.

Because of its various important functions in neurons [6, 8, 9, 27], RAB11 is considered indispensable for normal neuronal development. Indeed, gnomAD database showed that *RAB11A* gene was intolerant to loss-of-function variants, with a probability of being loss-of-function intolerant (pLI) score of 0.99 for *RAB11A* transcript (https://gnomad.broadinstitute.org/gene/ENSG00000103769?dataset=gnomad_r2_1). Transcripts with pLI scores ≥ 0.9 are considered intolerant to protein-truncating variations. Interestingly, however, we have identified one nonsense variant and one deletion variant resulting in a premature stop codon among gnomAD v3.1.2 (nonneuro) and v2.1.1 (nonneuro) datasets that essentially do not include data on individuals having neurological or psychological disorders according to dataset-selection guide in gnomAD (https://gnomad.broadinstitute.org/help/dataset-selection) (Figure 5). The variants, *c*.97*C* > *T* (p.(R33X)) and c.403delC (p.(P135Lfs^∗^22)) were identified in only a single allele, respectively, in the datasets and were extremely rare. Therefore, we should be cautious to conclude that the variations are unrelated to any disorders. At the moment, there is still insufficient information on the effects of loss-of-function, protein-truncating *RAB11A* variation on human development. On the other hand, all ID/DD-related *RAB11A* variants reported to date are missense and may be gain-of-function variants that adversely affect normal neurodevelopment. In fact, we have proven that the R33P variant associated with severe ID and hypomyelination is a constitutively active, gain-of-function variant. Among the disease-associated variants reported previously, only the K24R variant was proved to have promoted GEF activity probably due to its decreased affinity for both GTP and GDP [13]. Therefore, the K24R variant is supposed to be a constitutively inactive, another type of gain-of-function variant. It has not been determined whether each missense variant is constitutively active or inactive. Further biochemical and neurobiological studies on these variants are necessary to understand profound pathophysiological mechanisms underlying ID/DD associated with *RAB11A* variation.

The fact that the RAB11A-R33P is constitutively active and promotes neurite extension suggests that it is deleterious to normal neuronal development and functions, since proper axon growth and dendrite extension are the basis for the organization of complicated neuronal network and are spatiotemporally regulated. However, how it resulted in the hypomyelination and hypoplasia of the corpus callosum was enigmatic. Therefore, we focused on oligodendrocytes and confirmed that kinds of neuronal RABs including RAB11A are expressed also in oligodendrocytes. Furthermore, we found that the constitutively active Rab11A-Q70L promoted the outgrowth of processes in oligodendrocyte, and the constitutively inactive Rab11A-S25N attenuated it. These findings suggested that active RAB11A is a positive regulator of oligodendroglial development. The expression of the RAB11A-R33P caused excessive branching with thinner fine processes, which apparently differed from the case of the Q70L. It is unclear at the moment how the similarly constitutively active RAB11A-R33P and -Q70L affect oligodendroglial arborization differently. Considering that RAB11 functions through direct associations with the molecular motors as well as indirect associations with them mediated by various effector proteins, including RAB11-family interacting proteins (FIPs) in the regulation of endosome trafficking [1], one possible explanation is that the substitution interferes, not only in the GTP/GDP-bound status of the protein but also in interactions with such molecular motors and/or effector proteins. Although these multiple interactions are not fully understood, myosin V was proven to associate directly with the GTP-bound active form of RAB11 [28], while the dynein motor associates indirectly with RAB11 mediated by FIP3, one of RAB11-FIPs [29]. Intriguingly, the R33 as well as K13 predicted to be substituted in a previously reported case (case #3 in Table 2, Figure 5) is considered involved in the interaction with myosin V [30]. Furthermore, the R33 as well as R82 predicted to be substituted in another reported case (case #2 in Table 2, Figure 5) situates at the interface with FIP3 [31, 32]. On the other hand, Q70 was not assigned to the interaction with them, although it is inside the canonical effector recognition site [30]. Thus, the R33P variant is supposed to have altered affinities for Myosin V and/or FIP3, but the Q70L is not. Further substantial studies would be necessary to address the issue about the interaction between the RAB11A variants and various molecular motors and/or effector proteins. In any case, the formation of thinner excessive processes induced by the R33P would be detrimental to sound and effective myelination, since the numbers of myelinating processes extended from each oligodendrocyte are regulated properly *in vivo* [33]. We insist that this abnormally complicated arborization in oligodendrocytes is relevant to the hypomyelination observed in the present case. Since such hypomyelination seemed to be the common characteristics in ID/DD individuals harboring the DNM *RAB11A* variants, we suggest that studies about the previously reported *RAB11A* variants focusing on oligodendrocytes would be important. And further exploration into the dysfunctions in oligodendrocytes in association with unspecified, undiagnosed ID/DD would be necessary.

## 5. Conclusions

A novel *de novo RAB11A* variant, *c*.98*G* > *C* (p.(R33P)), identified in a boy with severe ID and hypomyelination, can induce abnormal morphogenesis in neurons and oligodendrocytes by generating a gain-of-function, constitutively active RAB11A. The findings strongly support the association between the *de novo* variation and neurodevelopmental impairments, especially the hypomyelination.

## Figures and Tables

**Table 1:** *In silico* analysis of *RAB11A* variants found in the present case and previously reported cases.

Variant	Domain	gnomAD v2.1.1	SIFT	PolyPhen-2	CADD PHRED	M-CAP	MutationTaster
*c*.98*G* > *C*, p.(R33P)	Small_GTP-bd_dom	−	0.02	0.994	27.4	0.149	1
*c*.39*A* > *C*, p.(K13N)^∗^	Small_GTP-bd_dom	−	0	0.987	33	0.787	1
*c*.71*A* > *G*, p.(K24R)^∗∗^	Small_GTP-bd_dom	−	0	0.979	27.1	0.673	1
*c*.244*C* > *T*, p.(R82C)^∗∗^	Small_GTP-bd_dom, switch II region	−	0	1.000	32	0.301	1
*c*.461*C* > *T*, p.(S154L)^∗^	Small_GTP-bd_dom	−	0	0.990	30	0.237	1
*c*.375*G* > *T*, p.(K125N)^∗∗∗^	Small_GTP-bd_dom	−	0	1.000	28.9	0.753	1
*c*.380*A* > *G*, p.(D127G)^∗∗∗^	Small_GTP-bd_dom	−	0	0.996	29.5	0.756	1

The variant found in our study is highlighted in gray. Locations of variants are annotated based on NM_004663.5. Small_GTP-bd_dom: small GTP-binding protein domain. ^∗^ Reported in reference [11], ^∗∗^ reported in reference [10], and ^∗∗∗^ reported in reference [12]. gnomAD v2.1.1 (http://gnomad.broadinstitute.org/) and SIFT (sorting intolerant from tolerant, https://sift.bii.a-star.edu.sg/): scores < 0.05 indicate that substitutions are predicted to be intolerant. Polyphen-2 (polymorphism phenotyping v2, http://genetics.bwh.harvard.edu/pph2/): scores are evaluated as 0.000 (most probably benign) to 0.999 (most probably damaging). CADD (combined annotation–dependent depletion, http://cadd.gs.washington.edu/score): PHRED scores of 10–20 and >20 are regarded as deleterious and the top 1% most deleterious, respectively. M-CAP (Mendelian clinically applicable pathogenicity, http://bejerano.stanford.edu/mcap/index.html): it correctly dismisses 60% of rare, missense variants of uncertain significance in a typical genome at 95% sensitivity. Scores of >0.025 are regarded as possibly pathogenic. MutationTaster (http://www.mutationtaster.org/): rapid evaluation of DNA sequence alterations. The alterations are classified as disease-causing or polymorphisms. Probability value is shown.

**Table 2:** Summary of the clinical features of individuals with DNM *RAB11A* variants.

Case	Gender	DNM variant	Cognitive and behavioral features	Brain MRI	Epilepsy	Associated neurological features	Miscellaneous features	Reference
#1	Male	*c*.71*A* > *G* p.(K24R)	GDD, moderate ID	Central brain atrophy, bilateral periventricular white matter damage, thin CC	None	Acquired microcephaly, axial hypotonia, aggressive behavior	Obesity	[10]

#2	Female	*c*.244*C* > *T* p.(R82C)	GDD, severe ID	Diffuse atrophy, partial agenesis of CC, delayed myelination	Infantile spasm	Acquired microcephaly, axial hypotonia		[10]

#3	NA	*c*.39*A* > *C* p.(K13N)	DD unspecified	NA	NA	NA		[11]

#4	Male	*c*.461*C* > *T* p.(S154L)	Moderate GDD	Partial agenesis of CC	None	Distractible, possibly ADHD		[11]

#5	Female	*c*.461*C* > *T* p.(S154L)	Moderate ID	ND	None	Possible hyperactivity	Obesity	[11]

#6	NA	*c*.375*G* > *T* p.(K125N)	GDD, ID	Agenesis of CC, hypomyelination	None	Microcephaly, nystagmus		[12]

#7	NA	*c*.380*A* > *G* p.(D127G)	ID	Agenesis of CC, abnormal myelination, abnormal cortical gyration	Seizure unspecified	Visual impairment	Dysmorphism	[12]

#8	Male	*c*.98*G* > *C* p.(R33P)	Severe ID	Thin CC, hypomyelination	None	Hypotonia		Present case

Abbreviations are as follows: ADHD: attention-deficit hyperactivity disorder; CC: corpus callosum; DD: developmental disorder; DDD: deciphering developmental disorders; DNM: de novo missense; GDD: global developmental delay; ID: intellectual disability; MRI: magnetic resonance imaging; NA: not available; ND: not done.

## Data Availability

The data that support the findings of this study are available from the corresponding author upon reasonable request.
